# Animal Models of Ascending Genital-Tract Infection in Pregnancy

**DOI:** 10.1155/S1064744994000414

**Published:** 1994

**Authors:** Robert S. McDuffie, Ronald S. Gibbs

**Affiliations:** 1 Department of Obstetrics and Gynecology Kaiser Permanente Denver CO USA; 2 University of Colorado Health Sciences Center Denver CO USA; 3 2045 Franklin Street Denver CO 80205-5494 USA

## Abstract

This article reviews animal models currently used for investigation of ascending genital-tract infection in pregnancy. The specific models reviewed are those in the rabbit, monkey, and mouse. These models investigate both the direct effects of bacteria in the setting of ascending infection and the role of cytokines produced by the immune system. For each model, experiments that delineate the pathophysiology of ascending genital-tract infection in pregnancy are described. Intervention experiments, including the use of antibiotics, anti-inflammatory agents, immunotherapy, and anti-cytokine therapy, are described. Comparison of these models is made with respect to pathogenesis in humans, reproducibility, anatomy, and cost.


Infectious Diseases in Obstetrics and Gynecology 2:60–70 (1994)
© 1994 Wiley-Liss, Inc.

### Animal Models of Ascending Genital-Tract Infection in Pregnancy

**Robert S. McDuffie, Jr., and Ronald S. Gibbs**

*Department of Obstetrics and Gynecology, Kaiser Permanente (R.S.M.) and University of Colorado Health Sciences Center (R.S.G.), Denver, CO*

ABSTRACT

**This article reviews animal models currently used for investigation of ascending genital-tract infection in pregnancy. The specific models reviewed are those in the rabbit, monkey, and mouse. These models investigate both the direct effects of bacteria in the setting of ascending infection and the role of cytokines produced by the immune system. For each model, experiments that delineate the pathophysiology of ascending genital-tract infection in pregnancy are described. Intervention experiments, including the use of antibiotics, anti-inflammatory agents, immunotherapy, and anti-cytokine therapy, are described. Comparison of these models is made with respect to pathogenesis in humans, reproducibility, anatomy, and cost.** © 1994 Wiley-Liss, Inc.

KEY WORDS

Intraamniotic infection, preterm labor, bacteria

In spite of concerted efforts over the past 2 decades to prevent premature delivery and low birth weight, these problems still represent the leading cause of perinatal morbidity and mortality in the United States. Traditionally, the major causes of preterm birth are categorized as 1) fetal and maternal indications, 2) premature labor, and 3) premature rupture of the membranes. There is ample evidence that infection, both clinical and subclinical, may lead to preterm delivery due to either premature labor or premature rupture of the membranes.[1] High rates of amniotic fluid (AF) infection, chorioamnionic infection, and histologic chorioamnionitis have been reported in low birthweight deliveries. These rates correlate inversely with gestational age of delivery. Many microorganisms and certain clinical infections have been associated with preterm labor, preterm delivery, and premature rupture of the membranes. Prostaglandins, arachidonic-acid metabolites, bacterial products, and cytokines have all been isolated from the chorioamnion, decidua or AF in cases of preterm birth, especially in those cases accompanied by infection. While this human evidence does build a strong case for the role of ascending infection in pregnancy as a cause of preterm birth, the case is largely circumstantial. Direct evidence is needed. Animal models in which variables can be controlled allow the hypotheses generated from human cases to be tested in the laboratory.

The purpose of this article is to review the current animal models of ascending infection in pregnancy. These models investigate the direct effects of bacteria in the setting of ascending infection, as well as the role of cytokines which are produced by the immune system. Particular emphasis will be given to the rabbit, monkey, and mouse models under current investigation.

### RABBIT MODEL

The rabbit has long been a model for bacterial infection. Advantages to this species have included

**Address correspondence/reprint requests to Dr. Robert S. McDuffie, Jr., Kaiser Permanente, 2045 Franklin Street, Denver, CO 80205-5494.**

Received March 25, 1994
Accepted June 16, 1994

Review Article

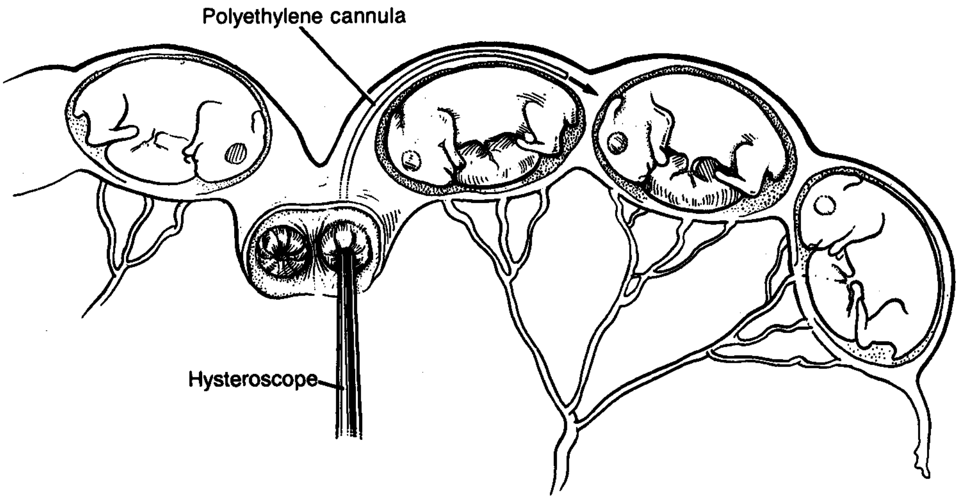

**Fig. 1. Schematic representation of experimental inoculation of rabbit uterine horns. (Reproduced from Dombroski et al.,[2] with permission of Mosby-Year Book, Inc.)**

not only its excellent response to microbiologic challenge, but also its size and ease of manipulation. Because of these latter attributes, direct bacterial challenge to the genital tract has been possible using a hysteroscope.

The rabbit is in a continuous state of estrus. Pregnancy can be achieved readily through natural means or through artificial insemination and ovulation induction by human chorionic gonadotropin. The gestational length is 30–31 days. The rabbit has 2 cervices and 2 uterine horns, similar to the human uterus didelphys. A thin vaginal septum separates the cervices. The average litter size is 7–8 pups.

### Experimental Model

Timed pregnant New Zealand white rabbits are anesthetized with xylazine hydrochloride (5 mg/kg) and ketamine hydrochloride (20–25 mg/kg). After perineal and lower vaginal preparation of the rabbit with povidone-iodine, a hysteroscope (Storz 27020B, Karl Storz Endoscopy-America, Inc., Culver City, CA) is introduced vaginally and advanced. The inoculum is delivered through the operating port of the hysteroscope via a sterile polyethylene cannula (0.15 cm in outer diameter) which is prefilled and attached to a tuberculin syringe. The bacterial inoculum may be placed in the uterus (Fig. 1), the cervix (Fig. 2), or the vagina. Once the inoculation is complete, the animal is returned to the cage for observation and/or treatment. Each inoculation can be completed within 3–5 min once the operator is experienced with the technique. Necropsy is performed after delivery or maternal death. At necropsy, cultures may be obtained from many sites, including the endometrium, AF, and blood; maternal and fetal serum and AF may be collected for study; tissues may be collected and fixed for histology.

### Organisms and Inoculation Site

Work in the rabbit model has evaluated the effect of different microorganisms, the level of inoculum placement in the genital tract, the natural history of the model, and intervention with antibiotics, anti-inflammatory agents, and passive immunization. Dombroski et al.[2] inoculated the uterine horn with *Escherichia coli*, *Fusobacterium necrophorum*, *Bacteroides bivius*, or sterile saline on day 21. High rates of clinical, microbiological, and histological infection were observed after bilateral intrauterine inoculation with both *E. coli* and *F. necrophorum* ($10^5$ cfu bilaterally). Animals inoculated with these organisms had high rates of fever (>40°C), delivery, and positive cultures of the AF. The mean time to delivery was approximately 30 h. None of the animals delivered live fetuses. Maternal deaths occurred in 3/7 rabbits inoculated with *F. necrophorum*. Histology confirmed an infiltration of polymorphonuclear leukocytes in the endometrium, amnion, and chorion after inoculation with *E. coli*. After inoculation with *F. necrophorum*, inflamma-

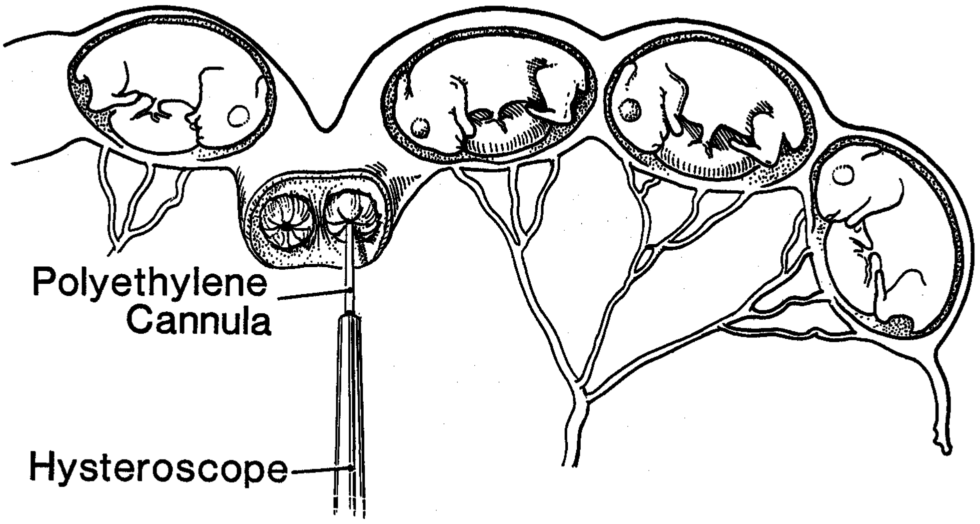

**Fig. 2.** Schematic representation of experimental inoculation of the rabbit cervix. (Reproduced from Heddleston et al.,[5] with permission of Mosby-Year Book, Inc.)

tion and necrosis were identified in the membranes and endometrium. An additional striking finding was the extensive placental abruption and engorgement of vascular channels within the placenta in animals inoculated with *F. necrophorum*.

In contrast, only a modest rate of clinical infection occurred after inoculation with *B. bivius* ($10^5$ cfu bilaterally). Although *B. bivius* was recovered from the endometrium in 22/29 (79%) animals, fever was observed in only 16% and live fetuses were seen in 88%. A low rate of infection was seen in control animals, largely due to contamination with enterococci. In the absence of inadvertent contamination, there was no clinical or histological evidence of infection in saline-inoculated animals.

In our laboratory,[3] we have confirmed the findings of Dombroski et al. for intrauterine inoculation with both *E. coli* and *F. necrophorum*. Further, in the absence of contamination, inoculation with pyrogen-free saline did not result in adverse maternal or fetal outcome. In all previous experiments, live bacteria had been used with bilateral uterine horn inoculation. We decided to test the effect of heat-killed organisms. On day 21, 5 animals were inoculated hysteroscopically into each uterine horn with 0.2 ml of $10^5$ cfu/ml of heat-killed *F. necrophorum* and observed for signs of delivery. Animals were observed up to 7 days after inoculation. Of the 5 animals inoculated with heat-killed organisms, 4 were put to death on day 7. These animals did not develop fever and did not deliver early, and all pups were live. One animal did have a positive culture for enterococcus in the AF and endometrium, but did not deliver. The fifth animal delivered 4 days after inoculation (day 25), but did not have a fever or positive culture. We concluded that we did not have a significant effect from the intrauterine inoculation of heat-killed *F. necrophorum*.

Since the previous experiments had all used bilateral intrauterine inoculation, we tested the effect of unilateral inoculation. We inoculated 3 animals with live *F. necrophorum* in only 1 uterine horn on day 21. Two of the 3 delivered prior to 7 days, and in both cases there was an associated maternal death. The third animal had no evidence of *F. necrophorum* at the time of necropsy, and all pups were live. From these pilot experiments, we have speculated that the overall outcome is not changed after unilateral uterine horn inoculation with *F. necrophorum*. Whether the mechanism of loss with unilateral inoculation results from a spread of infection to the contralateral horn or from the overwhelming effects of maternal sepsis remains unknown.

We have also performed experiments using intracervical inoculation with type-Ib group B streptococcus (GBS). Timed pregnant rabbits on day 21 were inoculated intracervically with $10^2$–$10^6$ of type-Ib GBS. Animals were put to death after heavy vaginal bleeding, delivery, or 6 days. Seven of 8 (88%) animals developed fever. All 8 animals delivered early, and a live fetus was present in only 1 of the litters. All 8 animals grew GBS from the endometrium; 5/7 (71%) from the AF; and 4/8 (50%) from maternal blood.

At the University of Texas, Field et al.[4] determined the effects of intrauterine inoculation of *Gardnerella vaginalis* on maternal and fetal outcome in the rabbit model. Animals on day 20–21 of gestation were inoculated hysteroscopically with $10^4$–$10^6$ cfu/ml of *G. vaginalis*. Animals were sacrificed on day 4 after inoculation, or earlier if delivery occurred. Of 17 animals inoculated with *G. vaginalis*, 3 developed fever, 2 had preterm labor, and 80% of the pups were born live. In addition, all 17 animals grew *G. vaginalis* from the decidua, and 15/17 from the AF. Peritoneal and blood cultures were negative in all 17 animals. In contrast, of 14 animals in the saline-inoculated group, none developed maternal fever, delivered early, or had positive cultures. Although differences between the 2 groups were not significant for most outcomes, there was a statistically significant decrease in the live-birth rate of animals inoculated with *G. vaginalis* compared with saline-inoculated animals (80% vs. 95%). Furthermore, when brain histology was performed, animals in the *G. vaginalis* group had a 60% incidence of severe brain injury compared with 0% in the saline-inoculated group. In summary, intrauterine inoculation with *E. coli* and *F. necrophorum* and intracervical inoculation with GBS resulted in clinical, microbiological, and histological evidence of infection. Intrauterine inoculation with *B. bivius* or *G. vaginalis* resulted in lower rates of clinical infection and fetal loss, but did result in high rates of positive cultures in the decidua. In the absence of contamination, saline inoculation did not result in any adverse clinical outcome.

The role of inoculum size has been studied in the rabbit model both for *E. coli* and GBS. Dombroski et al.[2] evaluated the effect of inoculum size with *E. coli* and found no difference in outcomes between inocula of $10^3$–$10^4$ and $10^7$–$10^8$ cfu/uterine horn. Using GBS in our laboratory, we observed high rates of fever and delivery after bilateral intracervical inoculation with $10^2$–$10^6$ cfu. Both of these virulent organisms produce high rates of infection independent of the inoculum size tested.

Because intrauterine inoculation with *E. coli* and *F. necrophorum* resulted in rapid intrauterine infection, fetal loss, and maternal sepsis, other inoculum sites were tested to determine the clinical course. In particular, for intervention experiments, it would be necessary to have a window of time prior to fulminant infection in which to test strategies for intervention. Direct intracervical placement offered the advantage of avoiding contamination of the sterile endometrium by vaginal bacteria. When Heddleston et al.[5] inoculated animals intracervically with *E. coli* (0.2 ml of $10^5$ cfu/ml), all 14 demonstrated fever within 24 h. Thirteen of 14 (93%) had bleeding at 24 h. Delivery by 24 h occurred in 7/14 (50%), and there were no live fetuses seen in any of the animals evaluated at delivery or at the time of necropsy. In contrast, in saline-inoculated animals, none had fever, bleeding, or delivery by 24 h. Live fetuses were found at necropsy in all cases, and there were no positive cultures.

In additional experiments in our laboratory, 6 animals were inoculated with $10^5$ cfu of *E. coli* in the upper vagina. Two of 6 ultimately developed clinical infection and aborted (1 mother died). Compared with intrauterine inoculation, intracervical inoculation has resulted in very similar outcomes except for a longer interval between inoculation and infection. As will be described later, this time allowed for antibiotic administration and pregnancy salvage.

### Role of Cytokines

The natural history of this model after intracervical inoculation with *E. coli* was described by McDuffie et al.[6] The primary objective of this study was to determine whether cytokines were produced in the AF after intracervical inoculation with *E. coli*. Timed pregnant rabbits on day 21 were inoculated intracervically with $10^4$–$10^5$ cfu of *E. coli*. Animals were put to death at 4, 8, 12, and 16 h after inoculation. Six control animals were put to death at similar intervals. Based on prior experiments, we knew that delivery and fetal loss occurred as early as 24 h. Therefore, we chose times in which we could measure cytokines before these outcomes occurred. Pooled AF was collected and assayed for tumor necrosis factor (TNF)-α bioactivity by a modified fibroblast cytotoxic assay using L929 cells. In addition, interleukin (IL)-1α, IL-1β, prostaglandin $E_2$, and prostaglandin $F_2\alpha$ were assayed by radioimmunoassay. Cultures were taken from the endometrium, AF, and blood. None of the control animals developed fever. None of 3 *E. coli*-inoculated animals sacrificed at 4 h had fever. Fever developed in 2/5 (40%) at 8 h, 2/3 (67%) at 12 h, and 4/5 (80%) at 16 h. Endometrial cultures were positive in all 16 *E. coli*-inoculated animals. AF cultures were positive in 1/3 animals killed at 4 h, 4/5 (80%) at 8 h, 3/3 (100%) at 12 h, and 4/5 (80%) at 16 h. Blood cultures were positive in 2/5 (40%) animals at 8 h, 3/3 (100%) at 12 h, and 4/5 (80%) at 16 h. Cultures were negative in all control animals. The AF levels of all 3 cytokines increased significantly with time from intracervical inoculation with *E. coli*. Similarly, levels of AF prostaglandin $E_2$ and prostaglandin $F_2\alpha$ increased significantly with time from inoculation with *E. coli*. In these studies, we demonstrated that TNF-α, IL-1α, and IL-1β are present in the AF after intracervical inoculation with *E. coli*. These studies confirm cytokine activation after microbiologic colonization of the decidua, since 0/6 control animals had detectable levels. Levels of cyotkines and prostaglandins were detectable as early as 4 h after intracervical inoculation and were detectable by 12–16 h after inoculation in all animals. The ascending nature of the model was confirmed again because AF cultures and blood cultures were positive with increasing frequency over time after intracervical inoculation.

### Intervention With Antibiotics

We also sought to determine whether intervention could improve the outcome of fulminant intra-amniotic infection and sepsis and fetal death which occurred uniformly after intrauterine inoculation. First, we studied the effect of antibiotic intervention after intrauterine inoculation with *E. coli*. Rabbits on day 21 or 22 were inoculated hysteroscopically with 0.2 ml of *E. coli* ($10^5$ cfu/ml). Animals received either no treatment or ampicillin/sulbactam, 100 mg/kg/day IM, in 3–4 divided doses, beginning 1–2 h before bacterial inoculation and continuing up to 7 days. An additional series of animals was inoculated with *E. coli* and treated with ampicillin/sulbactam immediately after inoculation (0 h, 2 h, or 4 h after inoculation). In this series, the dosage was increased to 150 mg/kg/day when serum ampicillin/sulbactam levels in the initial animals showed values in the low therapeutic range. As before, animals were killed after delivery or after 7 days. Of animals treated with ampicillin/sulbactam 1–2 h prior to inoculation, 3/11 (27%) delivered before 7 days, 3/11 had positive cultures, and at least 1 live fetus was present in all 11. These results were significant when compared with untreated animals, in which all 10 animals delivered and had positive cultures and no live fetus was present in any litter. In addition, the AF levels of prostaglandin $E_2$ and prostaglandin $F_2\alpha$ were significantly higher in untreated animals when compared with treated animals. The second series of animals, in which antibiotics were delayed for 2 and 4 h after inoculation, demonstrated that there was a very narrow window for antibiotic administration. As shown in Table 1, animals treated at 4 h after intrauterine inoculation with *E. coli* had significantly more deliveries, more positive cultures, and fewer live fetuses when compared with animals treated at or before the time of inoculation. Overall, there were improved outcomes in animals treated with ampicillin/sulbactam compared with those untreated; however, with intrauterine inoculation, there was a very narrow window for improving outcome.

**TABLE 1. Treatment with antibiotics: Outcome by *E. coli* intrauterine inoculum and time of treatment[a]**

| | Hours from *E. coli* inoculation to treatment[b] | | |
|---|---|---|---|
| Outcome | ≤0 | *P** | 4 |
| Delivery | 3/15 (20%) | <0.01 | 8/10 (80%) |
| Positive culture | 3/15 (20%) | <0.01 | 6/10 (60%) |
| Live fetus present | 15/15 (100%) | <0.001 | 2/10 (20%) |

[a]From McDuffie et al.[3]
[b]Ampicillin/sulbactam.
*Fisher's exact test.

We observed similar benefits in experiments using intrauterine inoculation with *F. necrophorum*. On day 21–22, rabbits were inoculated hysteroscopically with $10^4$–$10^5$ cfu of heat-killed or live *F. necrophorum*. Sixteen animals were inoculated with live *F. necrophorum* and randomly assigned to ampicillin/sulbactam or saline treatment beginning at the time of inoculation. Animals were put to death after delivery or after 6 days. As stated previously, experiments with heat-killed organisms did not result in significant pregnancy loss. Those rabbits treated with ampicillin/sulbactam had significantly more live fetuses, fewer positive cultures, and less fever at 24 h than those untreated (Table

**TABLE 2. Treatment with antibiotics: Outcome by intrauterine inoculum and treatment for *F. necrophorum***

| | | Live | | |
|---|---|---|---|---|
| Outcome | Killed | Untreated | P* | Treated[a] |
| Fever | 0/5 (0%) | 5/6 (83%) | <0.01 | 0/8 (0%) |
| Live fetus | 5/5 (100%) | 0/7 (0%) | <0.001 | 8/8 (100%) |
| Positive culture | 1/5 (20%) | 7/7 (100%) | <0.001 | 0/8 (0%) |

[a]Ampicillin/sulbactam, 150 mg/kg/day in 4 divided doses.
*Fisher's exact test.

2). Untreated animals had a clinical course of rapidly developing intraamniotic infection and maternal sepsis. Vaginal bleeding was observed in all 7 saline-treated animals by 24 h and in 0/8 antibiotic-treated animals.

### Intervention With Antibiotics and Indomethacin

Because of the short window of opportunity for treatment after intrauterine inoculation of *E. coli*, Heddleston et al.[5] conducted experiments with intracervical inoculation and delayed ampicillin/sulbactam therapy at 0, 4, 8, 12, and 16 h after inoculation with *E. coli*. Animals inoculated intracervically with *E. coli* and receiving delayed antibiotic therapy demonstrated gradually decreasing rates of fetal survival (Fig. 3). Animals inoculated intracervically that had antibiotics begun at 16 h had similar outcomes to animals inoculated with *E. coli* that were untreated. In an additional arm of this work, indomethacin pretreatment (0.2 mg/kg IV) was given to rabbits receiving no antibiotic therapy. In *E. coli*-inoculated animals receiving no antibiotic therapy, pretreatment with indomethacin significantly decreased bleeding and delivery within the first 24 h compared with those not pretreated, but did not improve overall fetal survival.

### Intervention With Type-Specific Antibody

In addition to antibiotic and anti-inflammatory intervention, the rabbit model had the potential for testing immunotherapy for prevention of experimental infection. Previous experiments by Larsen et al.[7] had suggested that passive immunization with modified immune serum globulin protected against rapid neonatal death in a monkey model. We have conducted preliminary experiments with passive immunization to type-Ib GBS in the rabbit. Twenty-four hours prior to inoculation, passive immunization with a polyclonal antibody to type-Ib GBS capsule polysaccharide (prepared in rabbits) was given in a dosage calculated to achieve a minimum of 2 μg/ml, which had been shown to be protective in other models. Rabbits on day 21 were inoculated intracervically with $10^2$–$10^6$ cfu of type-Ib GBS. Animals were put to death after heavy vaginal bleeding, delivery, or after 6 days. As seen in Table 3, there were no differences between treated and untreated groups in outcomes of fever, delivery, or presence of a live fetus. An assay for type-specific antibody concentration in maternal and fetal serum was not available.

### Summary

The rabbit model demonstrates rapid, reproducible intrauterine infection accompanied by maternal sepsis after inoculation. The most reproducible routes of inoculation are the direct placement into the endometrium or the cervix. There is evidence that high vaginal inoculation will produce modest rates of infection. For *E. coli*, *F. necrophorum*, and GBS, this infection appears to be an "all-or-none" phenomenon in that, once infection occurs, there is rapid progress to fulminant intraamniotic infection and sepsis. Intervention with antibiotics is effective within 0–2 h for intrauterine infection and up to 12 h after intracervical inoculation. Indomethacin pretreatment decreased fever in some treated animals, but did not result in improved clinical outcome. Limited experiments with passive immunization did not improve outcome. Other potential interventions with the model include comparative effects of antibiotics, use of cytokine modulators and blockers, and further work with immunotherapy.

### MONKEY MODEL

Primate models offer the desirable features of singleton gestation and uterine anatomy which are comparable to humans. Further, the placentation is hemochorial as in humans. The gestational length is approximately 160 days.

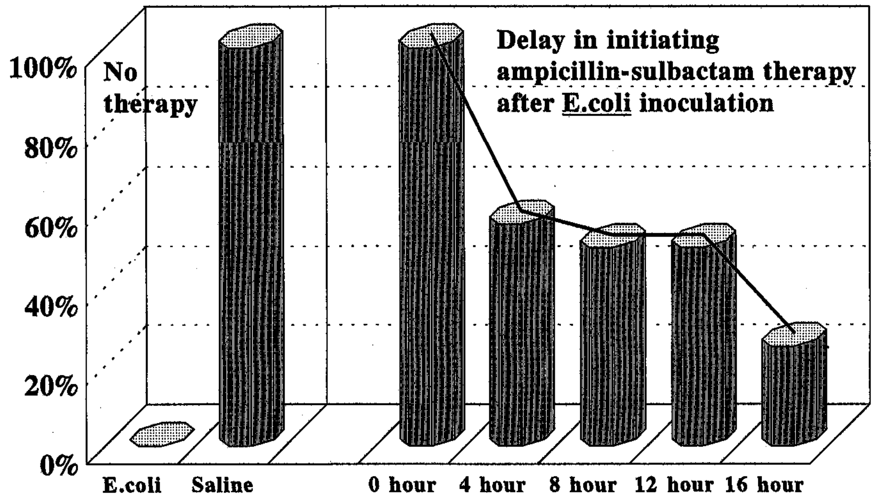

| # Rabbits | 25 | 6 | | 4 | 25 | 8 | 4 | 8 |
|---|---|---|---|---|---|---|---|---|
| Live fetus | 0 | 6 | | 4 | 14 | 4 | 2 | 2 |
| p-value * | - | <.001 | | <.001 | <.001 | .002 | .015 | NS |

Fig. 3. Fetal rabbit survival by delay in initiating antibiotic therapy after intracervical inoculation. *Fisher's exact test compared with *E. coli* and no therapy; NS = not significant. (Reproduced from Heddleston et al.,[5] with permission of Mosby-Year Book, Inc.)

TABLE 3. Treatment with type-specific antibody: Outcome by treatment group (intracervical inoculation of type-Ib GBS)

| Outcome | Untreated (N = 8) | *P** | Treated[a] (N = 11) |
|---|---|---|---|
| Fever | 7/8 (88%) | NS | 8/11 (73%) |
| Delivery | 8/8 (100%) | NS | 9/11 (82%) |
| Live fetus | 1/8 (12%) | NS | 3/11 (27%) |

[a]Type-specific antibody.
*Fisher's exact test; NS = not significant.

### Experimental Model

Timed pregnant rhesus monkeys *(Macaca mulatta)* undergo intraamniotic inoculation using percutaneous amniocentesis approximately 24 h prior to delivery (160 days). A 3.81-cm, 22-gauge needle is inserted through the abdominal wall in the midline above the umbilicus and then angled toward the pelvis in order to penetrate the uterus fundocephalad, since the usual attachment of the placenta is anterior. After placement is confirmed by checking for free aspiration of AF, a bacterial inoculum grown to logarithmic phase and diluted to the desired concentration is injected. Gross mixing of fluid and bacteria is performed by aspirating 5 ml of fluid and reinjecting the fluid 5 times. The animal is then returned to the cage for observation and/or treatment.

### Experimental Work

Larsen et al.[8] inoculated 10 rhesus monkeys intraamniotically near term with 0.5 ml of $10^5$–$2 \times 10^7$ cfu/ml of type-Ic or type-III GBS. No treatment was given in the initial series. Approximately 24 h after inoculation, the animals were delivered by cesarean section. Cultures were obtained from AF and cord blood at the time of cesarean section. In the neonate, peripheral blood cultures and spinal fluid were obtained 3 h after birth. At 24 and 48 h after birth, blood and cerebrospinal fluid cultures were taken from surviving neonates. Monkeys that died underwent prompt necropsy. Of animals inoculated with type-Ic GBS, 1/5 (20%) neonates died. Of animals inoculated with type-III GBS, 4/5 (80%) neonates died. GBS was consistently isolated from the AF, throat, and rectum in newborn monkeys. Nine of the 10 newborn monkeys had positive cord blood for GBS. Of the 5 animals that died, 1 was stillborn and the other 4 died between 25 and 96 h after inoculation (24 h prior to delivery). Spinal fluid cultures performed at 24 and 48 h of life were negative in survivors. Positive spinal fluid cultures occurred antemortum in the 2 monkeys that died at 96 h after inoculation. Viable colony counts in the AF reached $10^9$ cfu/ml at 24 h following inoculation. Necropsy showed pneumonitis in the 5 and meningitis in the 2 monkeys that survived for 96 h after inoculation. Since pneumonia was present in all monkeys that died after intraamniotic inoculation, aspiration of infected AF was likely an important factor in the development of disease. It was noted that fetal bacteremia, as manifested by positive cord blood culture, did not always predict outcome.

In an intervention trial, Larsen et al.[9] continued their work using antibiotic intervention immediately after intraamniotic inoculation. Nineteen pregnant rhesus monkeys at term were inoculated amniotically with approximately $10^6$ cfu of type-III GBS. Based on their previous work, type-III GBS was felt to be the more virulent organism. Inoculation occurred 24 h prior to cesarean delivery. Animals were treated with penicillin G, 20,000 U/kg IV, plus procaine penicillin G, 10,000 U/kg IM. Six of 10 untreated neonates died, while only 1/9 penicillin G-treated neonates died ($P = 0.04$) after intraamniotic inoculation with type-III GBS. Pneumonitis was discovered at autopsy in all neonates that died. In addition, 4/6 dead neonates in the untreated group had histologic evidence of meningitis. The neonate that died in the penicillin group had evidence of pneumonitis, but there was no meningitis; cultures were negative for type-III GBS. In the untreated control group, 7/10 (70%) neonates had positive cord blood cultures, 10/10 (100%) had positive throat cultures, and 3/10 (30%) had positive spinal fluid cultures. In the penicillin G-treated group, no neonate had a positive cord blood or spinal fluid culture. Three of 10 neonates in the treated group had a positive throat culture.

In further work, Larsen et al.[7] studied the relationship between susceptibility to intraamniotic infection with type-III GBS and concentration of type-specific antibody in serum of mothers and offspring. In 17 animals inoculated intraamniotically (controls), there was a significant association between the concentration of maternal antibody prior to infection and both the neonatal survival rate and survival time. In an intervention trial, modified immune serum globulin was given IV to 10 mothers prior to intraamniotic infection. Then, in their neonates, 5 animals received neonatal modified immune serum globulin and 5 did not. Although neither of the modified immune serum globulin groups demonstrated a reduction in neonatal mortality compared with controls, the authors concluded that this therapy provided protection against rapid neonatal death among those animals born to mothers with low antibody to type-III GBS prior to immunotherapy. As before, $10^6$ cfu of type-III GBS was inoculated intraamniotically 24 h prior to delivery. The concentration of antibodies of native polysaccharide antigen of type-III GBS was quantitated in sera by radioactive antigen-binding assay. Maternal sera were collected prior to administration of modified immune serum globulin and 24 h after administration. Neonatal sera were collected at birth. Modified immune serum globulin was given to mothers as an IV infusion of 5 ml/kg (250 mg/ml) over a 1-h period 24 h prior to intraamniotic infection. Neonates who received immune serum globulin received an IV injection of 5ml/kg in the umbilical vein over 3 min immediately after birth. In the control group, 12/17 (71%) neonates died. Levels of antibody to type-III GBS were lower in the preinoculation sera of mothers whose neonates died than of mothers of survivors. In the 2 groups treated with modified immune serum globulin, 3/5 (60%) neonates died in each group. This rate was not different from the rate of control animals. Antibody concentrations in the 22 paired maternal sera and cord sera were compared. A significant correlation was seen between antibody concentrations in maternal sera collected prior to delivery and matched neonatal cord sera, which indicated antibody transfer in most instances. In this study, in untreated animals, a statistically significant relationship between the concentration of maternal antibody and neonatal survival was demonstrated.

Ten years later, Gravett et al.[10] used the monkey model to establish a relationship between AF infection, cytokines, prostaglandins, and uterine activity. In 3 chronically instrumented rhesus monkeys at day 130 of gestation, AF infection was established by intraamniotic inoculation of $10^6$ cfu of type-III GBS. In this chronically instrumented model, maternal blood, fetal blood, and AF were sampled sequentially. Increases in uterine contractility occurred between 14 and 36 h after inoculation in all 3 monkeys and led to progressive cervical dilation in 2/3 monkeys. Sequential sampling of AF for bacteria, IL-1β, and prostaglandins revealed increases in bacterial count from a preinoculation rate of 0 to $10^9$ cfu/ml at delivery and increases in AF IL-1β, prostaglandin $E_2$, and prostaglandin $F_2\alpha$ over time from inoculation. Further, it was felt that increases in cytokines and prostaglandins began prior to increases in contractility.

### MOUSE MODEL

The mouse offers another model for infection in pregnancy, with a short gestational length (19–21 days) and low cost. However, experimental manipulation, such as direct intrauterine or intracervical inoculation, amniocentesis, or chronic catheterization, is more difficult. Nevertheless, the mouse has been the subject of many reports and historically preceded other animal models for infection in pregnancy. The majority of these studies did not strictly study ascending infection, but rather used bacterial products (endotoxin) or cytokines to induce preterm labor, pregnancy loss, and occasionally maternal death.

In 1943, Zahl and Bjerknes[11] studied the effects of gram-negative endotoxin in a pregnant mouse model. They injected 150 mice intraperitoneally with *Shigella* endotoxin at 12, 15, and 18 days of gestation. By 8 h after injection, nearly all animals experienced bleeding. No living fetuses were born from any of the mothers. The predominant mode of pregnancy loss was hemorrhage and abruption, which led either to labor and expulsion of the embryos or to fetal resorption. Takeda and Tsuchiya[12] prepared an endotoxin from *E. coli* that was injected IV into pregnant mice before 20 days of gestation. These animals were then observed for 24 h. In all cases, the animals aborted and fetal death occurred. In addition, the maternal death rate was 75%. More recently, Gendron et al.[13] examined the phenomenon of lipopolysaccharide-induced fetal resorption in mice because Zahl and Bjerknes[11] had demonstrated that bacterial endoxotins induced fetal resorption (in addition to causing abortion). IV administration of 25 μg of lipopolysaccharide on day 12 resulted in the appearance of TNF-α in the AF and fetal resorption. In addition, they performed intervention studies using pentoxifylline, a drug known to suppress formation of TNF-α. They demonstrated that levels of lipopolysaccharide-induced TNF-α were reduced by 90% after pretreatment and concluded that TNF-α is involved in lipopolysaccharide-induced fetal resorption in mice.

In other recent studies, Romero et al.[14] studied the role of IL-1 in preterm parturition in mice to determine whether systemic administration of IL-1 could induce parturition in mice. Twenty-four mice were randomized to receive human recombinant IL-1α or sterile phosphate-buffered saline subcutaneously at 15–17 days of gestation. Injections were performed using 1 μg of IL-1α diluted in 100 μl of phosphate-buffered saline. Three injections were given over 5.5 h. No animals receiving phosphate-buffered saline exhibited vaginal bleeding. In IL-1α-treated animals, vaginal bleeding was noted as early as 2 h after the second injection and by 12 h after commencement of the protocol. Ten of 12 (83%) animals had vaginal bleeding. Preterm delivery occurred in 11 IL-1α-treated animals (1 animal died). All animals in the control group delivered at 20 days or later. Live fetuses were seen in all 12 control animals.

In later experiments, Romero and Tartakovsky[15] determined whether pretreatment with the IL-1 receptor antagonist could prevent preterm parturition induced by IL-1α. Animals were randomly allocated to an initial set of experiments in which the authors determined the dose of IL-1 that would reliably induce preterm delivery after subcutaneous administration. In the second phase, animals were treated with human recombinant IL-1 and increasing dosages of IL-1 receptor antagonist. They did demonstrate that a combination of human recombinant IL-1 (10 μg) and IL-1 receptor antagonist (1 mg) administered subcutaneously did not result in premature delivery in 12 pregnant mice. Using a similar dose of IL-1, lower dosages of IL-1 receptor antagonist (0.25 and 0.5 mg) were associated with increased rates of premature delivery (100% and 66.6%, respectively).

In summary, studies on the mouse have provided insight regarding the systemic administration of bacterial products, cytokines, and cytokine blockers. In this paradigm, systemic administration results in a local effect on the uterus, preterm labor. In contrast, ascending infection causes release of cytokines and prostaglandins within the uterus and intrauterine infection prior to systemic spread of disease. Therefore, it is uncertain whether the mouse model, which uses systemic administration to achieve effect, truly mimics ascending infection in pregnancy.

### DISCUSSION

Each of these 3 models offers different observations on infection in pregnancy. It is important now to compare and contrast these models with respect to several issues. First and foremost, the model must mimic the pathogenesis in humans. Second, the model must be reproducible. Further, it is important to discuss the applicability of the animal model to the human state. Other important issues include cost, anatomy, and pharmacokinetics.

Ascending infection is accepted as the mode of bacterial entry to the uterus in cases of intraamniotic infection. There is strong circumstantial evidence that subclinical infection is linked to premature birth.[1] In the models presented, only the rabbit model mimics natural ascending infection in pregnancy. By hysteroscopic placement, a variety of organisms can be directly placed into the uterus, cervix, or vagina. The level of placement directly correlates with the reproducibility of infection. With *E. coli* or GBS, nearly 100% of animals inoculated in either the uterus or cervix develop clinical infection. However, when the inoculum is placed in the upper vagina, a lower percentage of animals develop clinical disease. Generally, the organism placed is the organism subsequently recovered with clinical infection. The exception to this is the occasional enterococcal infection seen with intrauterine inoculation. In our laboratory, the enterococcus is cultured as normal flora from the rabbit vagina. Work in the monkey model bypasses the cervix and upper vagina via direct bacterial placement in the amniotic cavity. This step provides certainty that the bacterial inoculum will be placed where fetal effect can occur. The ascending nature of the rabbit work is further confirmed by increasing rates of positive cultures over time, including AF and maternal blood cultures in the natural history data.

An additional piece of evidence for ascending infection came from experiments in which antibiotic intervention occurred 0–4 h after intrauterine inoculation with *E. coli.* In this experiment, in the cases where live animals were found after antibiotic treatment, they were always found in the distal horns, suggesting that the antibiotic had had effect prior to bacteria ascending to the distal horns. In contrast, the mouse model did not use live bacteria and instead focused on bacterial products and cytokines which, when administered systemically, led to fetal death and/or abortion.

The disease state produced in the rabbit is that of intraamniotic infection leading to maternal sepsis. In the monkey model, neonatal pneumonitis, meningitis, and sepsis are documented. While the disease states produced are important when applied to humans, it is disappointing that a model more reminiscent of subclinical infection and preterm labor has not been produced. The work by Field et al.[4] demonstrates that a chronic intrauterine infection with *G. vaginalis* can be established, but the rate of preterm labor is not increased significantly over the rate of saline-inoculated animals. Perhaps trials with other organisms may produce different results.

We are able to show that antibiotic intervention after intrauterine and intracervical inoculation results in improved clinical outcome, although there is a very narrow window of time in which it occurs. As in intraamniotic infection in humans, levels of prostaglandins and cytokines are elevated after bacterial infection. This is confirmed in both the rabbit and monkey models. Because of technical considerations, it is necessary to pool AF in the rabbit model in order to obtain enough for several assays. In the monkey, there is a greater amount of AF present. This potentially could be harvested by both repeated aminocenteses or chronic catheterization, as done by Gravett et al.[10] The advantage of this latter technique allows for serial sampling within a given individual. It is more difficult in small animals to achieve the technology necessary to sample serially.

Clearly, the underlying physiology favors the monkey model because of its comparability to humans. Both uterine anatomy and litter size are markedly different for both the rabbit and the mouse. In addition, maternal pharmacokinetics and the pharmacokinetics of placental transfer may be markedly different from species to species. The cost factor obviously favors a less-expensive model with a shorter gestation, as in the rabbit and mouse models. Nevertheless, the work in the monkey is critical to the validity of other work obtained from less-costly models.

In this review, we have discussed the current animal models of ascending infection in pregnancy. The primary advantages of the rabbit model are its ascending nature, reproducibility, low cost, and applicability to the human situation. The advantages of the monkey model are its close relation to humans and the size of the animal, which allows for serial sampling and improved neonatal information. The disadvantages of the monkey model include its cost and long gestation. The mouse model is the least costly of the models. The effects of parenteral administration of bacterial products and cytokines in the mouse mimic the end stage of ascending infection in which there is a systemic maternal response. Nevertheless, the administration of cytokines has produced preterm parturition in mice. The role of true ascending infection in this model remains unexplored. These models all shed light on the evolving picture of infection as a cause of preterm birth. Future studies will continue to elucidate mechanisms of preterm labor associated with infection in these animal models.

### REFERENCES

1. Gibbs RS, Romero R, Hillier SL, Eschenbach DA, Sweet RL: A review of premature birth and subclinical infection. Am J Obstet Gynecol 166:1515–1528, 1992.
2. Dombroski RA, Woodard DS, Harper MJK, Gibbs RS: A rabbit model for bacteria-induced preterm pregnancy loss. Am J Obstet Gynecol 163:1938–1943, 1990.
3. McDuffie RS, Blanton SJ, Shikes RH, Gibbs RS: A rabbit model for bacterially induced preterm pregnancy loss: Intervention studies with ampicillin-sulbactam. Am J Obstet Gynecol 165:1568–1574, 1991.
4. Field NT, Newton ER, Kagan-Hallet K, Peairs WA: Perinatal effects of *Gardnerella vaginalis* deciduitis in the rabbit. Am J Obstet Gynecol 168:988–994, 1993.
5. Heddleston L, McDuffie RS, Gibbs RS: A rabbit model for ascending infection in pregnancy: Intervention with indomethacin and delayed ampicillin-sulbactam therapy. Am J Obstet Gynecol 169:708–712, 1993.
6. McDuffie RS, Sherman MP, Gibbs RS: Amniotic fluid tumor necrosis factor-α and interleukin-1 in a rabbit model of bacterially induced preterm pregnancy loss. Am J Obstet Gynecol 167:1583–1588, 1992.
7. Larsen JW, Harper JS, London WT, Baker CJ, Curfman BL, Kasper DL, Sever JL: Antibody to type III group B *Streptococcus* in the rhesus monkey. Am J Obstet Gynecol 146:958, 1983.
8. Larsen JW, London WT, Palmer AE, Tossell JW, Bronsteen RA, Daniels M, Curfman BL, Sever JL: Experimental group B streptococcal infection in the rhesus monkey. Am J Obstet Gynecol 132:686–690, 1978.
9. Larsen JW, London WT, Baker CJ, Curfman BL, Sever JL: Intraamniotic infection due to group B *Streptococcus:* Treatment and antibody response. Obstet Gynecol 58: 222–226, 1981.
10. Gravett MG, Baggia S, Haluska GJ, Cook MJ, Novy MJ: An experimental model for preterm labor associated with amniotic fluid infection in rhesus monkeys. Abstract No. 11, Annual Meeting of the Infectious Disease Society for Obstetrics and Gynecology, South Hampton, Bermuda, August 1991.
11. Zahl PA, Bjerknes C: Induction of decidua-placental hemorrhage in mice by the endotoxins of certain gram-negative bacteria. Proc Soc Exp Biol Med 54:329–332, 1943.
12. Takeda Y, Tsuchiya I: Studies on the pathologic changes by the injection of the Shwartzman filtrate and the endotoxin into pregnant animals. Jpn J Exp Med 23:105–110, 1953.
13. Gendron RL, Nestel FP, Lapp WS, Baines MG: Lipopolysaccharide-induced fetal resorption in mice is associated with the intrauterine production of tumour necrosis factor-alpha. J Reprod Fertil 90:395–402, 1990.
14. Romero R, Mazor M, Tartakovsky B: Systemic administration of interleukin-1 induces preterm parturition in mice. Am J Obstet Gynecol 165:969–971, 1991.
15. Romero R, Tartakovsky B: The natural interleukin-1 receptor antagonist prevents interleukin-1-induced preterm delivery in mice. Am J Obstet Gynecol 167:1041–1045, 1992.
